# Supplementary material for: Seismic performance of base-isolated structures for swimming pool reactors under different foundation conditions
Source: PLoS One. 2024 Oct 18;19(10):e0311216. doi: 10.1371/journal.pone.0311216 (PMC11488743; doi:10.1371/journal.pone.0311216)
Supplement: Attachment — Submitted filename: Response to Reviewers.docx [file pone.0311216.s002.docx]

Title:	Seismic performance of base-isolated structures for swimming pool reactors under Different foundation conditions

Authors:	Jie Zhao, Jianshan Wang, Jiehua Huang

Paper No.:	PONE-D-24-23674

**Dear Editor,**

The authors would like to thank the reviewers for their valuable comments and suggestions. The reviewers’ comments have all been taken into consideration in this revised manuscript. The authors believe that these suggestions significantly improve the quality of this paper. The following presents our replies to the questions and concerns that are raised by the Reviewers.

**======================================================**

**Reply to Reviewer 1’s Comments:**

1. Please, revise some typos in the text and figures (e.g., Figure 1).
**Reply:** Agreed, thank you for the expert's comments. The revisions have been made in the manuscript.

2. Within Introduction and text, discuss that a numerical model is always affected by two families of uncertainties (i.e., aleatory and epistemic), as discussed in:
Gino, D. et al., Strain-based method for assessment of global resistance safety factors for NLNAs of reinforced concrete structures, Engineering Structures, 2024, 304, 117625.
Miceli, E. et al., Approaches to estimate global safety factors for reliability assessment of RC structures using non-linear numerical analyses, Engineering Structures, 2024, 311, 118193.
**Reply:** Agreed, thank you for the expert's comments. The main research focus of this paper is the seismic performance study of base isolation structures for pool-type reactors, and the developed viscoelastic boundary has been validated through case studies, proving its accuracy. The expert's suggestions are very helpful, and I will consider the two types of uncertainties in numerical models (i.e., aleatory and epistemic) more in future research.

3. Please, provide numerical details (e.g., criteria convergence).
**Reply:** Agreed, thank you for the expert's comments. The force convergence criterion for dynamic calculations is 0.05, and the displacement convergence criterion is also 0.05. The soil is modeled using the Mohr-Coulomb model, the concrete structure is considered linear elastic. The paper employs a direct coupling method for fluid and solid, enforcing the equality of degrees of freedom at coincident nodes on the fluid-solid interface to achieve displacement transfer.

4. Comments if the concrete confinement effects are considered, as discussed in:
Miceli, E., et al., Confinement effects within the seismic design of reinforced concrete frames: reliability assessment and comparison, Engineering Structures, 2024, 313, 118248.
**Reply:** Agreed. Thank you for the expert's comments. The paper does not consider the specific constraint effects of concrete structures, which is a very good suggestion. I will consider the concrete confinement effects of concrete structures in future research.
5. Please, provide the inherent damping factors used in the numerical analyses.
**Reply:** Agreed. Thank you for the expert's comments. According to Standard for Seismic Design of Nuclear Power Plants, the recommended inherent damping factor is 5%.

6. Discuss if the records can be considered frequent or no-frequent ground motions as well as their PGA/PGV ratios, as described in:
Castaldo, P., Miceli, E. Optimal single concave sliding device properties for isolated multi-span continuous deck bridges depending on the ground motion characteristics, Soil Dynamics and Earthquake Engineering, 2023, 173, 108128.

**Reply:** Agreed. Thank you for the expert's comments. Currently, there are no specific seismic design standards internationally. Therefore, the Standards for the Evaluation of Seismic Safety of Nuclear Power Plants are followed. The seismic waves used in this paper are the improved version of the Regulatory Guide 1.60 seismic design response spectrum developed by the US Nuclear Regulatory Commission in 1973, with a PGA value of 0.3g.

**Reply to Reviewer 2’s Comments:**

1. The comparison of first-order natural vibration frequency and natural vibration period of the structures after isolation with different conditions (i.e., 1 to 4) is not different. As a result, it is expected the similar response of isolated structure with different conditions.

**Reply:** Agreed. Thank you for the expert's comments. The modification has been made in line 298 of the manuscript.

2. It is not clear what the authors are conveying through “diagonal increase” on line 311 of the manuscript.

**Reply:** Agreed. Thank you for the expert's comments. The “diagonal increase” in the manuscript means that both the structural height and the structural acceleration are increasing simultaneously.

3. Some more details about the elements used in the ANSYS model for the analysis shall be provided.

**Reply:** Agreed. Thank you for the expert's comments. In the numerical model analysis, SOLID185 elements were used to simulate the structure and foundation. Based on single-node definition, the software's built-in structural mass element MASS21 was chosen for simulation. For the effects of liquid in the water storage tank and spent fuel pool, FLUID80 elements were used based on the Housner theory, with coupled degrees of freedom used to simulate fluid-structure interaction effects. The foundation was divided into uniform grids as much as possible, with structural element sizes ranging from 0.5 to 2 meters, foundation element sizes shall not exceeding 5 meters. The model consists of 88,031 solid elements, 7,488 fluid elements, and 39 concentrated mass elements, totaling 95,558 elements and 111,171 nodes.

4. Authors must explain about the User Programmable Features (UPFs) and FORTRAN, subprograms written in FORTRAN interface with ANSYS to customize user elements (refer to lines 133-134).

**Reply:** Agree, thanks to the expert's comments. the User Programmable Features (UPFs) serve as a supplement and extension to the APDL’s functionality. Essentially, it is a form of source code-level secondary development. Through a series of custom files (user subroutines) provided by ANSYS, users can edit and modify them, then use specific versions of compilers (MS C++ and FORTRAN) to generate a customized version of ANSYS software. This allows users to achieve desired functions or capabilities not provided by the software, such as optimization algorithms, user-defined failure criteria, and contact criteria, as well as the development of complex material constitutive models.

5. The authors shall provide More details about the selected earthquake ground motions such as year, components, PGA, etc.

**Reply:** Agreed, and thank you for the expert's comments. Currently, there are no specific seismic design standards for this internationally. Therefore, The seismic waves used in this paper are the improved version of the Regulatory Guide 1.60 seismic design response spectrum developed by the US Nuclear Regulatory Commission in 1973, for earthquake response analysis based on the seismic safety review standards for nuclear power plants. The corresponding response spectrum is a broad-band spectrum that encompasses various damping conditions and is conservatively designed to typically envelop the response spectra of specific or expected sites in the country, providing a good characterization of the seismic capacity for nuclear facilities. The total duration is 28 seconds with a time step of 0.01 seconds. The PGA values are 0.3g in the X and Z directions, and 0.2g in the Y direction.

6. Some results shall be presented considering the real and recorded near-fault earthquake ground motions.

**Reply:** Agree, thank you for the expert's comments. This paper uses the seismic design response spectrum for nuclear power plants. The expert's advice is excellent, and I will consider real and recorded near-fault earthquake motions in future research and present some results.

7. The model of the base-isolated structure (refer to Figure 1) in the present study considers the flexibility of the superstructures and ignores the impact at the base on the adjacent moat wall. The above assumption for the selected model shall be justified by citing suitable papers like https://doi.org/10.1155/2003/368693 , https://doi.org/10.1177/1077546309103271 , and others.

**Reply:** Thank you for the expert's comments. Figure 1 is a schematic representation of the static and dynamic models of viscoelastic artificial boundaries. According to the comments of experts, the base isolation structure has been improved in line 243.

8. The presentation of the paper needs to be further improved to meet international journal standards.

**Reply:** Agree, thank you for the expert's comments. The entire manuscript has been polished and improved.

Please feel free contact us if further revision is needed.

Thank you very much for your attention.

Best regards,

Dr. Zhao

School of Architectural Engineering

Dalian University

Dalian 116622

P. R. China

E-mail: zhaojie_gd@163.com
