# Supplementary material for: Seismic performance of base-isolated structures for swimming pool reactors under different foundation conditions
Source: PLoS One. 2024 Oct 18;19(10):e0311216. doi: 10.1371/journal.pone.0311216 (PMC11488743; doi:10.1371/journal.pone.0311216)
Supplement: Attachment — Submitted filename: Response to Reviewers.docx [file pone.0311216.s003.docx]

Title:	Seismic performance of base-isolated structures for swimming pool reactors under Different foundation conditions

Authors:	Jie Zhao, Jianshan Wang, Jiehua Huang

Paper No.:	PONE-D-24-23674R1

**Dear Editor,**

The authors would like to thank the reviewers for their valuable comments and suggestions. The reviewers' opinions have been considered in the revised manuscript, and four additional references have been added according to their suggestions. The authors believe that these recommendations have significantly improved the quality of the paper. Below are our responses to the questions and concerns raised by the reviewers.

**======================================================**

**Reply to Reviewer 1’s Comments:**

1. Within Introduction and text, discuss that a numerical model is always affected by two families of uncertainties (i.e., aleatory and epistemic), as discussed in:
Gino, D. et al., Strain-based method for assessment of global resistance safety factors for NLNAs of reinforced concrete structures, Engineering Structures, 2024, 304, 117625.
Miceli, E. et al., Approaches to estimate global safety factors for reliability assessment of RC structures using non-linear numerical analyses, Engineering Structures, 2024, 311, 118193.
In detail, comment how the both uncertainties can influence the results and which one can have a dominant role.

**Reply：**Thank you for the expert's opinion. In numerical model analysis, there are always two types of uncertainties: aleatory uncertainty (i.e., materials, geometric properties and actions) and epistemic uncertainty (i.e., modeling). Aleatory uncertainty arises from the inherent randomness in data or models. For example, there may be measurement noise in the experimental data, or there may be random disturbances in the simulation. Epistemic uncertainty arises from incomplete knowledge of the model or system, including simplification, approximation, or inaccuracy of assumptions. For example, simplification of physical models, roughness of computational grids, etc. By improving the model, increasing computational accuracy, and reducing assumptions, cognitive uncertainty can be reduced. This article is dominated by epistemic uncertainty, and the correctness of viscous-spring boundary is verified through numerical examples to reduce epistemic uncertainty. Within Introduction and text, discuss that a numerical model is always affected by two families of uncertainties (i.e., aleatory and epistemic), and cited four relevant literature, including two articles recommended by the reviewers. The opinions of experts are very forward-looking, and I will focus on studying aleatory and epistemic uncertainty in future research

2. Discuss if the records can be considered frequent or no-frequent ground motions as well as their PGA/PGV ratios, as described in:
Castaldo, P., Miceli, E. Optimal single concave sliding device properties for isolated multi-span continuous deck bridges depending on the ground motion characteristics, Soil Dynamics and Earthquake Engineering, 2023, 173, 108128.
In detail, provide the value of the ratio “PGA/PGV” discussing if it is a high or medium or low value.

**Reply：**The expert's opinion is very good. When using PGA/PGV for long structures such as long-span cable-stayed bridges, there will be significant changes in the displacement response of the structure. The structure of this article is a pool type reactor, not a long structure. According to the "Code for Seismic Design of Buildings" and "Seismic Design Standards for Nuclear Power Plants", the seismic parameters used are mostly the peak ground acceleration (PGA) and acceleration response spectrum. This article records seismic waves that cannot be considered frequent or infrequent. The seismic waves used are an improved version of the 1.60 Seismic Design Response Spectrum developed by the US Nuclear Regulatory Commission in 1973, which is used for seismic response analysis based on the seismic safety review standards for nuclear power plants. The corresponding response spectrum is a broadband spectrum that covers various damping conditions and is conservatively designed to typically cover specific or expected locations in the country, thus well describing the seismic capacity of nuclear facilities. The total duration is 28 seconds, with a time step of 0.01 seconds. The PGA value is 0.3g in the X and Z directions and 0.2g in the Y direction. There is a detailed explanation on line 287. Thank you for your interesting feedback. In future research, we will consider the impact of seismic parameters using PGA/PGV on the structure.

Please feel free contact us if further revision is needed.

Thank you very much for your attention.

Best regards,

Dr. Zhao

School of Architectural Engineering

Dalian University

Dalian 116622

P. R. China

E-mail: zhaojie_gd@163.com
